# Orbital Mucormycosis: Understanding the Deadly Fungus Sweeping the Globe

**DOI:** 10.7759/cureus.41010

**Published:** 2023-06-26

**Authors:** Shaheen Farooq, Nabab A Khan, Ajeet Singh, Arif Khan, Preeti Sharma, Ritu Meena, Ankita Jakhar, Mukesh Kumar, Anju Kochar

**Affiliations:** 1 Ophthalmology, Sardar Patel Medical College, Prince Bijay Singh Memorial (PBM) Hospital, Bikaner, IND

**Keywords:** retrobulbar amphotericin b, steroids, diabetes mellitus, covid-19, mucormycosis

## Abstract

Introduction

Mucormycosis (black fungus) is a rare opportunistic fungal infection commonly affecting immunocompromised individuals. There has been a surge in the number of these cases during the second wave of the coronavirus disease 2019 (COVID-19) in India. Mucormycosis has been reported to occur within a week or a few weeks post-recovery from COVID-19. The most common clinical manifestation of mucormycosis is rhino-orbital-cerebral mucormycosis (ROCM). At our tertiary care center, we initiated a prospective study to identify risk factors, study ocular manifestations, and explore medical and surgical management of orbital mucormycosis patients in the post-COVID-19 era.

Material and methods

This is a detailed description of a prospective observational hospital-based study. The study included 148 patients who presented with ROCM. A detailed history was taken regarding the complaint, duration, and associated risk factors. Systemic, local, and complete ophthalmic examinations were done that included assessment of extraocular movements, visual acuity, slit-lamp examination, and fundus examination. All data were recorded separately for each patient in a pre-decided proforma.

Result

The study group consisted of 148 patients. In our study, the highest association was with COVID-19-positive status (68.24%), out of which 57 (56.43%) were on oxygen support. Diabetes mellitus contributed next to COVID-19 with 86 (58.10%) patients with a positive history of diabetes. Seventy-one (47.97%) patients were on steroids, out of which 68 (67.32%) were COVID-19-positive and the rest (23%) were on steroids due to various systemic reasons. Rhinomaxillary involvement was present (51%). Out of 63 patients with orbital involvement, 16 (25.39%) presented bilaterally and 47 showed unilateral orbital involvement more on the right side (42.85%). The predominant location of orbital involvement was the orbital apex. The most common symptom seen in our study was nasal discharge (86.5%), and ophthalmoplegia was the most common sign.

Conclusion

Corticosteroids should be used with caution to prevent negative impact and potential ROCM. Good glycemic and metabolic control is crucial for treatment. Management of mucormycosis involves surgical debridement, antifungal agents, and retrobulbar amphotericin B injections. Early diagnosis and aggressive treatment are essential for success. Orbital exenteration may be necessary for advanced stages, while conservative approaches may work for earlier stages. Patient counseling is needed for cosmetic rehabilitation. A multidisciplinary approach involving various specialists is necessary.

## Introduction

The world's healthcare system was already grappling with the catastrophic coronavirus disease 2019 (COVID-19) pandemic when the emergence of mucormycosis, a fungal infection, further burdened it. Mucormycosis was previously observed mainly in immunocompromised patients, such as those with uncontrolled diabetes mellitus, hematologic malignancies, and other similar conditions [1]. However, the second wave of COVID-19 witnessed a surge in cases of this hidden fungal infection across the globe.

The most common clinical manifestation of mucormycosis is rhino-orbital-cerebral mucormycosis (ROCM), which starts with inhalation of spores, can spread to the sinuses, adjacent tissues such as the sphenoid sinuses, and orbits, and eventually reaches the brain. This infection is characterized by tissue necrosis, which appears as a black eschar or discharge in the nasal or oral cavity, hence the name "black fungus" [2,3].

The nonspecific symptoms of the disease may cause a delay in diagnosis, and rapid progression is responsible for the high mortality rate of 40%-80%. Hence, early diagnosis remains the most significant challenge and is essential to reduce morbidity and mortality [4].

The increased incidence of ROCM in an already overwhelmed healthcare system posed various challenges to clinicians, including ophthalmologists, who struggled with diagnosing and managing this condition. Due to the rarity of mucormycosis historically, research has been limited, and studies were conducted across the globe to develop management guidelines and strategies to prevent and combat this infection.

At our tertiary care center, we initiated a prospective study to identify risk factors, study ocular manifestations, and explore medical and surgical management of orbital mucormycosis patients in the post-COVID-19 era. We also aim to study the outcome and long-term follow-up of patients treated for this condition, which has been a significant limitation in previous research articles.

## Materials and methods

This study describes a prospective observational hospital-based study conducted on patients with mucormycosis at a tertiary care center. The study included 148 patients who presented with ROCM or were histologically/potassium hydroxide (KOH) smear positive for mucormycosis between May 1, 2021, and April 30, 2022. The study was conducted after obtaining permission from the Institutional Ethics and Research Board of Sardar Patel Medical College with approval number F.29(Acad)SPMC/2023/2024, and informed consent was obtained from each patient in their language. A detailed history was taken regarding the complaint and duration. History regarding risk factors including immunocompromised status such as a history of COVID-19 infection, diabetes, immunosuppression, steroid intake, oxygen therapy, and previous antibiotic therapy was elicited in detail with leading questions and their previous records if present.

The examination involved assessing the nasal cavity, oral cavity, orbit, and cranial nerves. A local ocular examination was conducted, which included evaluating the orbital margins, extraocular movements, lids, conjunctiva, cornea, anterior chamber, pupil, and iris using a slit lamp. Visual acuity was measured using the Snellen chart. Routine investigations were performed, including complete blood count, renal function test, liver function test, urine analysis, HIV and HBsAg screening, chest X-ray, and electrocardiogram (ECG). Diabetic patients underwent fasting and post-prandial blood glucose evaluation. Diagnostic nasal endoscopy was performed for all patients, and the findings were recorded. Samples were collected for histopathological examination, either through a swab for KOH smear or tissue biopsy, to confirm the diagnosis of mucormycosis. Only patients with confirmed mucormycosis were included in the study. Radiological investigations included high-resolution computed tomography (CT) of the paranasal sinuses, orbit, and brain, as well as magnetic resonance imaging (MRI) of the brain, based on the patient's clinical condition. All investigations were documented in a proforma.

The study primarily focused on patients with orbital mucormycosis. The disease was staged according to the clinical extension, following the staging proposed by the Collaborative OPAI-IJO Study on Mucormycosis in COVID-19 (COSMIC) study group (Appendix). Rhinomaxillary mucormycosis involved the maxilla, oral cavity (palate), retromaxillary space, pterygopalatine fossa, and infratemporal fossa. Rhino-orbital mucormycosis included patients with orbital (intraconal and extraconal) lesions, preseptal cellulitis, and orbital cellulitis extending to the orbital apex, with or without maxillary involvement. Rhino-orbital-cerebral mucormycosis included patients with cavernous sinus and intracranial region involvement, with or without orbital and maxillary involvement.

The management approach was individualized for each patient. Medical management included strict glycemic control, correction of metabolic factors, control/reversal of immunocompromised conditions such as neutropenia, renal parameter correction, anemia correction if necessary, and antifungal medication. Expert opinions were sought from various specialties, including diabetology, nephrology, ENT, neuro-ophthalmology, neurology, neurosurgery, hematology, dentistry, general medicine, mycology, microbiology, and pathology. The main drug used in this study was intravenous amphotericin B. It was administered at a dose of 1 mg/kg body weight/day, up to a maximum of 50 mg/day, infused with 5% dextrose over 4-6 hours after initially hydrating the patient with 500 mL of normal saline. Liver function tests were closely monitored during treatment. Retrobulbar amphotericin B was given in selected patients under all aseptic precautions. A 1.5-inch-long 26-gauge needle was used to deliver 1 mL of 3.5 mg of liposomal amphotericin B (L-AMB) to the retrobulbar space, with injection directed toward the region of radiographic disease in the orbit. Gentle pressure was applied to the eyes, and patients were observed for five minutes after injection to monitor for signs of orbital compartment syndrome and reassessed after four hours of the procedure. Patients received once a day until day 5 and thereafter on alternate days, and patients were then reassessed for the disease status. Surgical procedures included endoscopic debridement (functional endoscopic sinus surgery (FESS)) and exenteration. All patients were followed up for one year, with reviews at one month, six months, and one year. Complete ocular examinations were conducted at each visit, and computed tomography of the orbit was performed if necessary.

## Results

In this study, the mean age of the population was 49.14±16.27 years. Out of 148 patients, 91 (61.48%) were males and 57 (38.51%) were females with a male/female ratio of 1.59:1.

The risk factors associated are tabulated in Table 1. The highest proportion was with coronavirus disease 2019 (COVID-19)-positive status (101, 68.24%), out of which 57 (56.43%) were on oxygen support. Diabetes mellitus (type 2) contributed next to COVID-19 (86, 58.1%), with 54 (36.48%) patients having a positive history of diabetes and 32 (22.85%) newly diagnosed. Seventy-one (47.97%) patients were on steroids, of which 68 were COVID-19-positive, and the rest (3) were on steroid therapy for various systemic reasons.

**Table 1:** Various risk factors associated with mucormycosis *Chronic liver disease, COPD, and chronic alcoholism COVID-19: coronavirus disease 2019, COPD: chronic obstructive pulmonary disease

Risk factor	Number of patients	Percentage
COVID-19 status	101	68.2
Steroid use	71	47.9
Oxygen use	57	38.5
Diabetes mellitus	86	58.1
Chronic sinusitis	1	0.7
Hypertension	15	10.1
Chronic kidney disease	3	2.02
Heart surgery	1	0.7
Immunosuppressive	2	1.4
Organ transplant	1	0.7
Malignancy	2	1.4
Chronic disease*	3	2

The maximum number of patients presented to us in stage 2, where rhinomaxillary involvement was present (51%). Stage 3 with orbital involvement was seen in 40 (27%) patients, which further increased to 63 (42.56%) due to the disease progression of patients of stage 1 and stage 2, as shown in Table 2.

**Table 2:** Stage of ROCM at presentation ROCM: rhino-orbital-cerebral mucormycosis

Stage of ROCM	Number of patients	Percentage
ROCM 1	12	8.2
ROCM 2	76	51.35
ROCM 3	40	27.2
ROCM 4	20	13.6
Total	148	100

Out of 63 patients with orbital involvement, 16 (25.39%) presented bilaterally, and 47 showed unilateral orbital involvement more on the right side (42.85%). The predominant location of orbital involvement was the orbital apex, as shown in Table 3.

**Table 3:** Orbital involvement predominant location

Orbital involvement predominant location	Number of cases	Percentage
Medial orbit	4	6.34
Superior orbit	2	3.2
Interior orbit	13	21
Apex	43	69.4
Diffuse	1	1.6
Total	63	100

The clinical signs and symptoms observed in our study are depicted in Table 4 and Table 5. The most common symptom seen in our study was nasal discharge (86.5%), and ophthalmoplegia was the most common sign.

**Table 4:** Ocular sign CRAO: central retinal artery occlusion

Ocular sign	Number of cases	Percentage
Lid edema	15	10.1
Corneal ulcer	1	0.7
Conjunctival chemosis	40	20
Ptosis	48	32.4
Orbital cellulitis	15	10.1
Optic neuritis	46	31.1
CRAO	16	10.8
Ophthalmoplegia	54	36.5

**Table 5:** Primary symptoms

Primary symptom	Number of cases	Percentage
Nasal block	68	45.9
Nasal discharge	128	86.5
Orbital/facial pain	123	83.1
Orbital/facial edema	67	45.3
Orbital/facial discoloration	9	6.1
Ptosis	48	32.4
Diplopia	0	-
Proptosis	42	28.4
Diminution of vision	60	40.5

In this study, IV amphotericin B was the mainstay of treatment given in all 148 patients. Renal functions and serum electrolyte levels were closely monitored. Retrobulbar amphotericin B was given to 26 patients classified as ROCM stage 3 with minimal orbital disease, as shown in Table 6. Out of 15 patients exenterated, two were stage 4, 10 were stage 3d, and three were stage 3c.

**Table 6:** Management of mucormycosis FESS: Functional endoscopic sinus surgery

Medical management	Number of cases	Percentage
IV amphotericin B	148	100
Retrobulbar amphotericin B	26	17.6
Surgical management	Number of cases	Percentage
FESS	145	97.9
FESS+exenteration done	15	15.1
Repeat FESS	7	4.7

The outcome of the disease with the stage of presentation and final vision outcome are depicted in Table 7 and Table 8, respectively. Out of 15 exenterated patients, three deaths were reported: two were stage 4 patients and one was stage 3d.

**Table 7:** Outcome of disease with the stage of presentation ROCM: rhino-orbital-cerebral mucormycosis

ROCM stage	Outcome			Total	Statistics (p value)
	Good	Death	Survival rate		
ROCM 1	8 (5.7%)	0 (0%)	100%	8 (5.7%)	0.007*
ROCM 2	38 (27.1%)	9 (6.4%)	80.9%	47 (33.6%)	
ROCM 3	43 (30.7%)	20 (14.3%)	68.3%	63 (45%)	
ROCM 4	10 (7.1%)	12 (8.6%)	45.5%	22 (15.7%)	
Total	99 (70.7%)	41 (29.3%)	70.7%	140 (100%)	

**Table 8:** Final vision outcome PL: perception of light, PR: projection of rays

Final vision outcome	First month (n=115)	Sixth month (n=110)	12th month (n=102)
PL negative	14 (29.2%)	13 (28.9%)	12 (27.9%)
PR inaccurate	10 (20.8%)	8 (17.8%)	7 (16.3%)
PL present to 6/60	22 (45.8%)	22 (48.9%)	22 (51.2%)
6/60 to 6/18	2 (4.2%)	2 (4.4%)	2 (4.7%)
Better than 6/18	0 (0%)	0 (0%)	0 (0%)
Total	48 (100%)	45 (100%)	43 (100%)

We lost 25 (16.89%) patients during the treatment, and eight (5.40%) left against medical advice. One hundred four patients were discharged on tablet posaconazole 800 mg/day for one month. Eleven (7.43%) patients had no signs and symptoms on discharge and were declared cured of ROCM (Table 9).

**Table 9:** Discharge status LAMA: leave against medical advice

Discharge status	Number of cases	Percentage
Cured	11	7.4
LAMA	8	5.4
Under treatment	104	70.3
Death	25	16.9
Total	148	100

The final outcome at follow-up is shown in Table 10. Twenty-five patients were lost to death on discharge. On follow-up, 16 more patients died, as shown in Table 10. The total number of reported deaths is 41.

**Table 10:** Outcome at follow-up

Outcome at follow-up	First month (n=115)	Sixth month (n=110)	12th month (n=102)
Alive with regression	68	78	83
Alive with stable residual	20	15	10
Alive with progression	22	9	6
Death	5	8	3

## Discussion

Our research focused on rhino-orbital-cerebral mucormycosis (ROCM), an emerging threat associated with coronavirus disease 2019 (COVID-19), which has become a significant concern in healthcare facilities worldwide. We studied 148 patients who presented with ROCM during the second wave of the COVID-19 pandemic. The majority of the cases were between 51 and 60 years old, with a decrease in extremes of age. The mean age was 49.14±16.27 years [5-8].

Mucormycosis is rarely encountered in the pediatric age group. In the pediatric epidemiological study of 63 patients by Pana et al. [9], the disseminated form (38%) was the most common type, and hematological malignancy (46%) was their most common underlying condition. In our study, we had only one child with acute lymphoblastic leukemia (ALL). In most studies, there was a male predominance, possibly due to greater outdoor exposure to fungal spores [5-7,10].

The mainstay of management of COVID-19 was largely supportive care. The use of steroids played a pivotal role too. During the second wave of COVID-19, ROCM became highlighted and strongly associated with COVID-19. Our study found that 68.24% of ROCM cases were COVID-19-associated, out of which 56.43% were on oxygen support, and 47.97% were on steroids, out of which 67.32% were COVID-19-positive [11].

The use of glucocorticoids in patients with COVID-19 has several effects, including increasing blood glucose levels, antagonizing macrophage maturation and differentiation, suppressing the microbicidal activity of activated macrophages, inhibiting neutrophil chemotaxis, lysosomal enzyme secretion, and respiratory burst [12]. Three patients in our study were on chronic steroid therapy, and steroids were temporarily withdrawn, and these patients responded well. However, the practical or indiscriminate use of corticosteroids can cause a negative impact and may be a possible cause of ROCM. Hence, caution is warranted.

Diabetes mellitus contributed next to COVID-19, with 86 (58.10%) patients having a positive history of diabetes [13]. Adequate glycemic and metabolic control, thus reversing the underlying condition, is one of the prime principles of the treatment strategy [14].

In our study, hypertension was present in 15 (10.13%) patients. Nineteen (12.83%) patients were immunocompromised for various reasons such as malignancy, chronic kidney disease (CKD), organ transplant, heart surgery, and immunosuppressive treatment.

**Figure 1:**
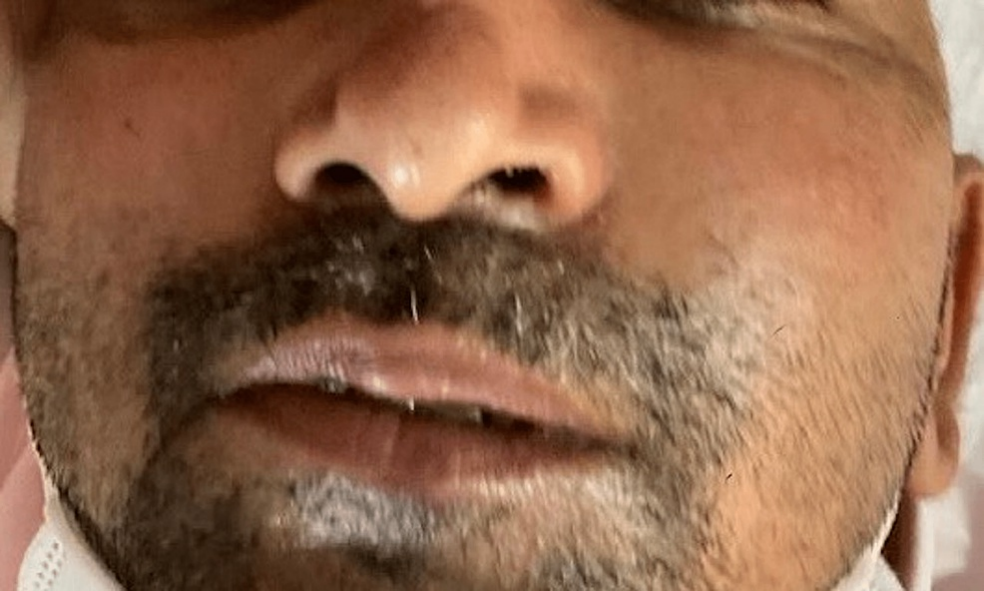
Facial edema

**Figure 2:**
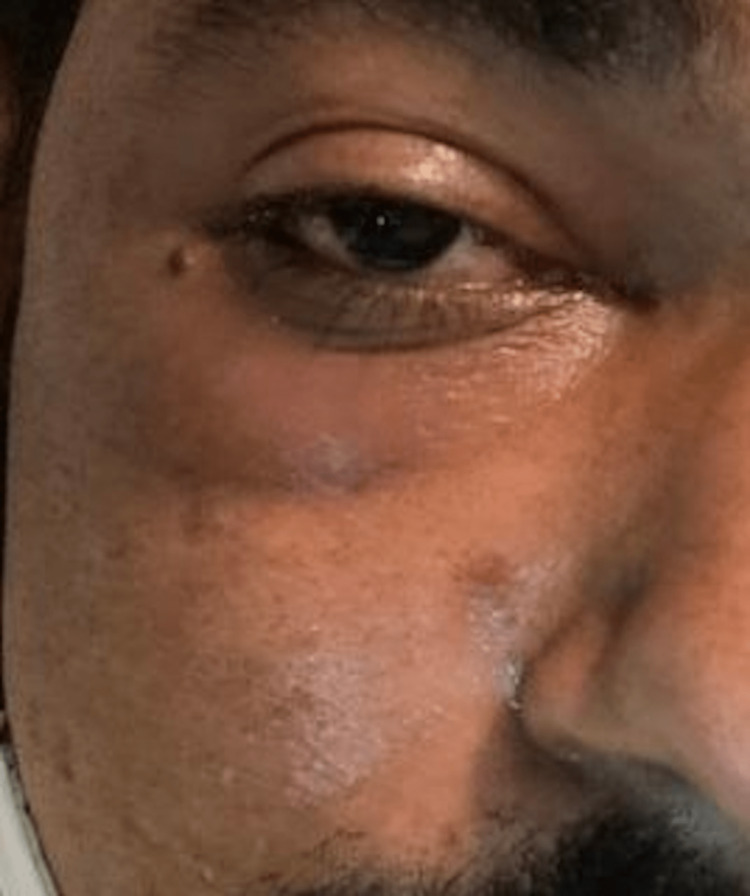
Facial and periorbital edema

**Figure 3:**
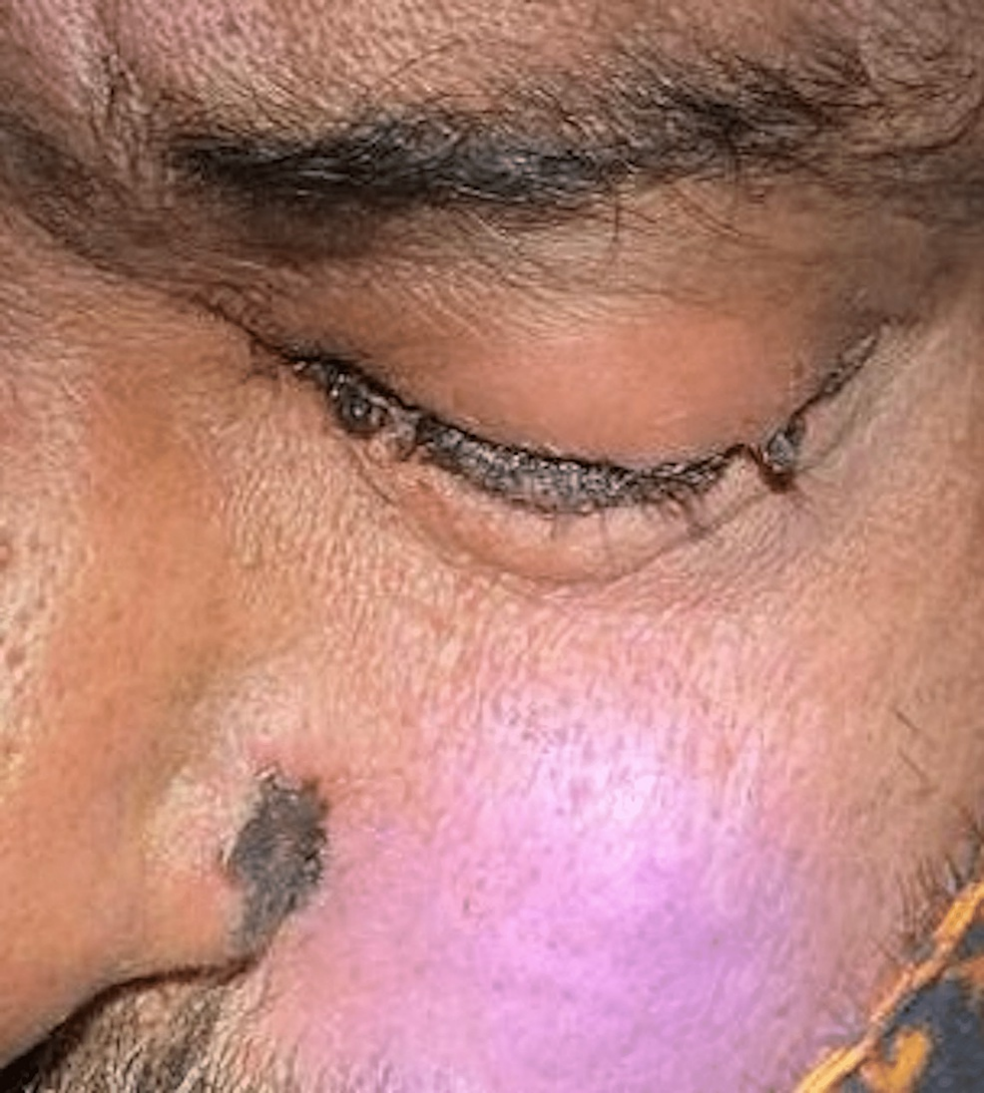
Facial edema and discoloration of the face

**Figure 4:**
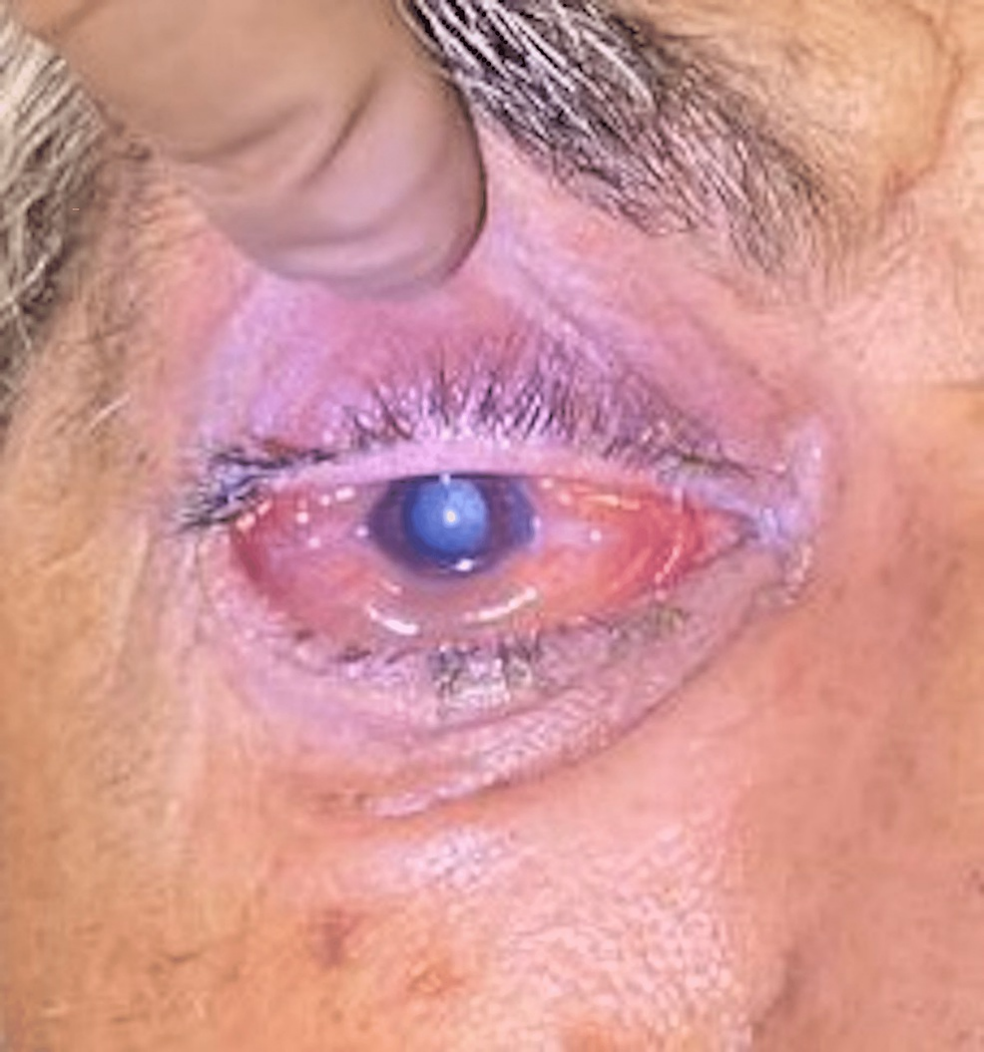
Conjunctival chemosis and lid edema

**Figure 5:**
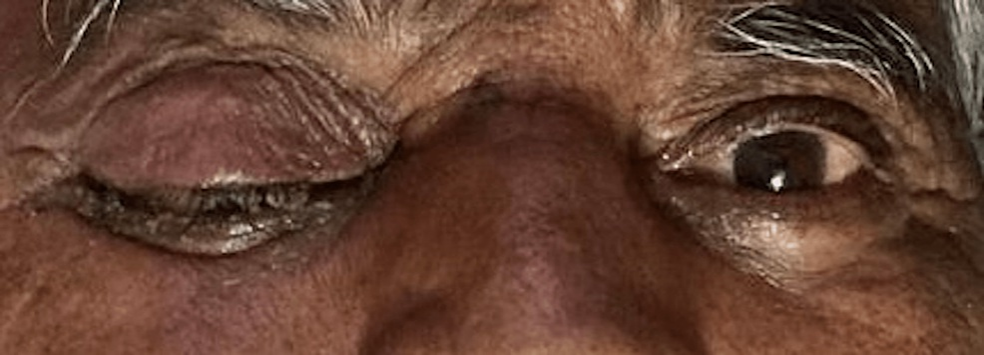
Drooping of the eyelid

**Figure 6:**
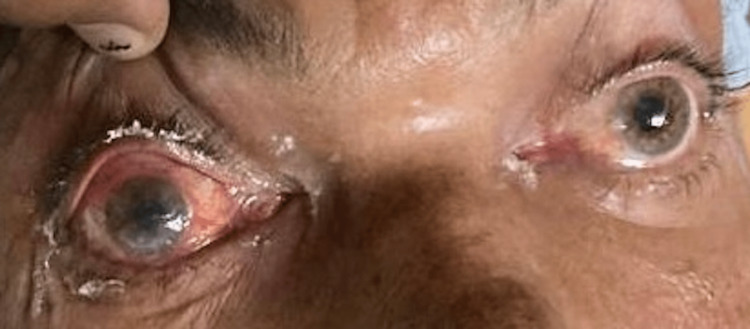
Proptosis in the right eye

The most common signs and symptoms reported by the COSMIC study were decreased vision, orbital/facial pain, periocular/facial edema, ptosis, and nasal discharge [10]. The most common symptoms seen in our study were nasal discharge (86.48%), followed by orbital/facial pain (83.10%). Ocular symptoms were orbital/facial edema (45.27%) (Figures 1-4), diminution of vision (40.54%), drooping of the lid (32.43%) (Figure 5), and proptosis (28.37%) (Figure 6).

**Figure 7:**
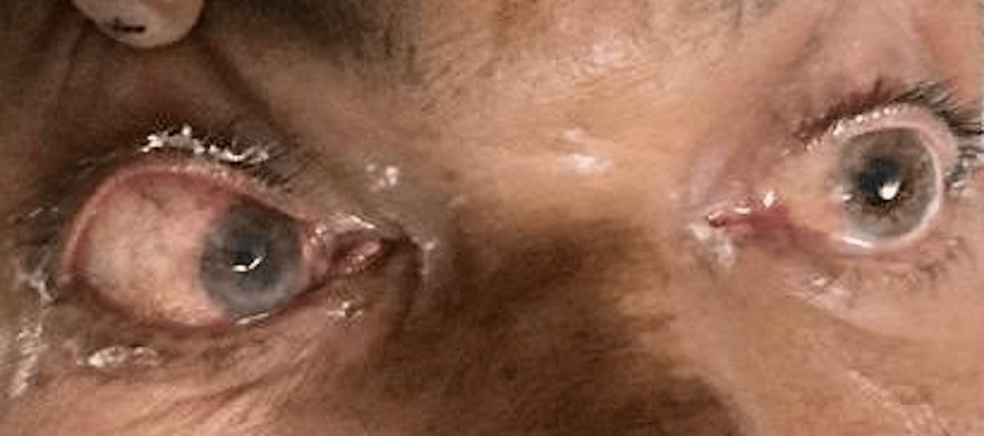
Complete ophthalmoplegia in the left eye

We did not encounter anyone complaining of diplopia in our study. Ophthalmoplegia (Figure 7) was the most common sign seen in 54 patients, followed by ptosis in 48 patients.

**Figure 8:**
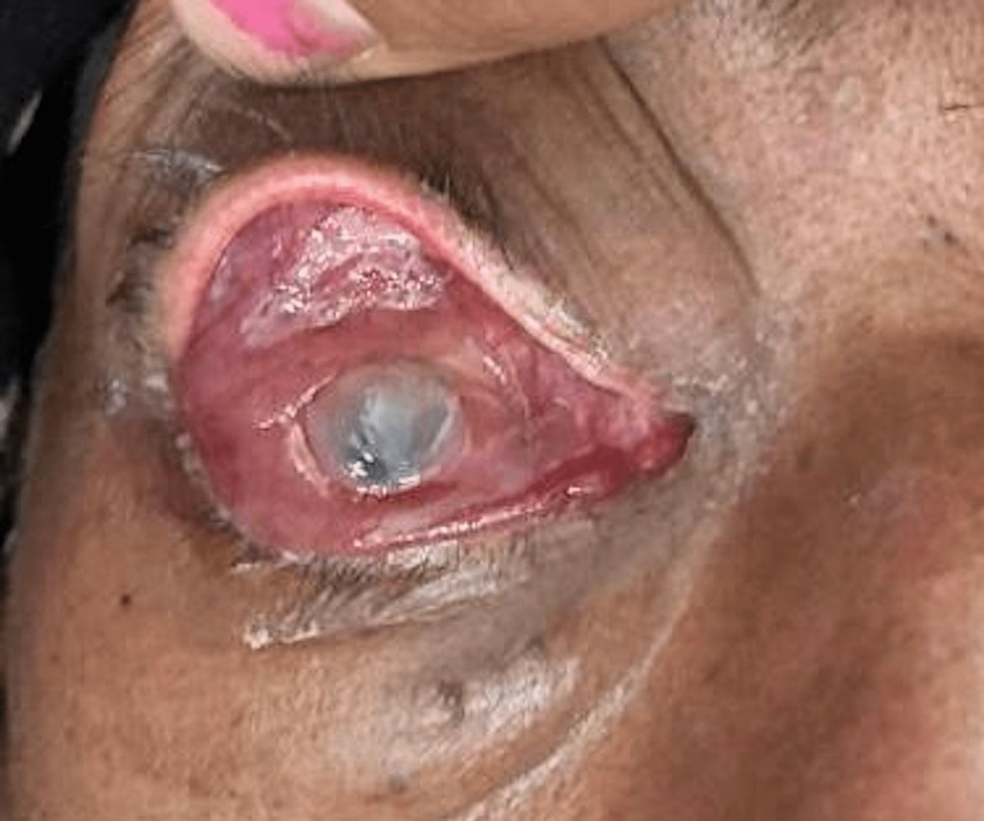
Exposure keratopathy due to proptosis

Optic neuritis was noticed in 46 patients. Sixteen patients had central retinal artery occlusion. One patient had a corneal ulcer attributed to exposure keratopathy due to proptosis (Figure 8).

The orbital apex was predominantly involved in 68.25% of the patients with ocular involvement (n=63). Early diagnosis of apical orbital involvement may minimize the risk of progression to the cavernous sinus. However, in the COSMIC study, the medial wall was predominantly involved. CNS involvement has been documented in 37% of cases of COVID-19-associated ROCM [10]. In our study, 14% of patients had CNS involvement, whereas, in the COSMIC study, CNS involvement was seen in 21% of patients [10].

The diagnosis was based on reported clinical and preclinical features of COVID-19-associated mucormycosis. The combination of CT scans and MRIs helps the physician diagnose more accurately and evaluate the extension of involvement.

Functional endoscopic sinus surgery (FESS) was performed in 97.97% of cases. It served as a therapeutic and diagnostic tool for the suspected cases in stage 1 and early stage 2. For the rapid diagnosis of mucormycosis, direct microscopy using KOH wet mounts, culture to identify species, and antifungal susceptibility and histopathology for the definitive diagnosis of mucormycosis is required [15,16]. Polymerase chain reaction (PCR) is a rapid test compared to histopathology and should be made more widely available [17,18].

Managing mucormycosis involves the control of risk factors, surgical debridement, and antifungal agents. An extensive review of 929 cases showed that survival was only 3% with no intervention, 57% with surgery alone, 61% with amphotericin deoxycholate, and 70% when treated with both amphotericin and surgical debridement [10]. Amphotericin B is the antifungal drug of choice for mucormycosis. In our study, 100% of the patients had received liposomal amphotericin B (induction dose: 5 mg/kg body weight for stages 1a-3d and up to 10 mg/body weight for stages 4a-4d). One can use amphotericin B deoxycholate or amphotericin B lipid complex in resource constraints. The liposomal form can be given in higher doses and longer duration as it is less nephrotoxic. Prolonged step-down oral antifungal therapy is recommended for 3-6 months [18-20].

One hundred four (70.27%) patients received step-down treatment with posaconazole 800 mg/day. Step-down therapy with posaconazole has shown promising results, with complete resolution in 67% of patients [21]. Data regarding the efficacy and safety of combination therapy need to be improved in the literature, with no available guidelines [18-20].

Clinicians face challenges in deciding when to perform orbital exenteration for patients with orbital mucormycosis resistant to all medical and surgical interventions and progressing with intracranial involvement. There are no standard guidelines for this decision. Few studies found insignificant differences in survival with or without orbital exenteration [22,23]. Others have documented it to have a negative impact on survival. COSMIC study data showed that in patients with limited sino-orbital disease (stages 3a and 3b), orbital exenteration does not significantly alter the outcome [10]. However, when it advances to stages 3c and worse, orbital exenteration helps improve the outcome. The analysis of the data of our study also confirmed this. We exenterated patients with stage 3c and 3d and stage 4 ROCM.

**Figure 9:**
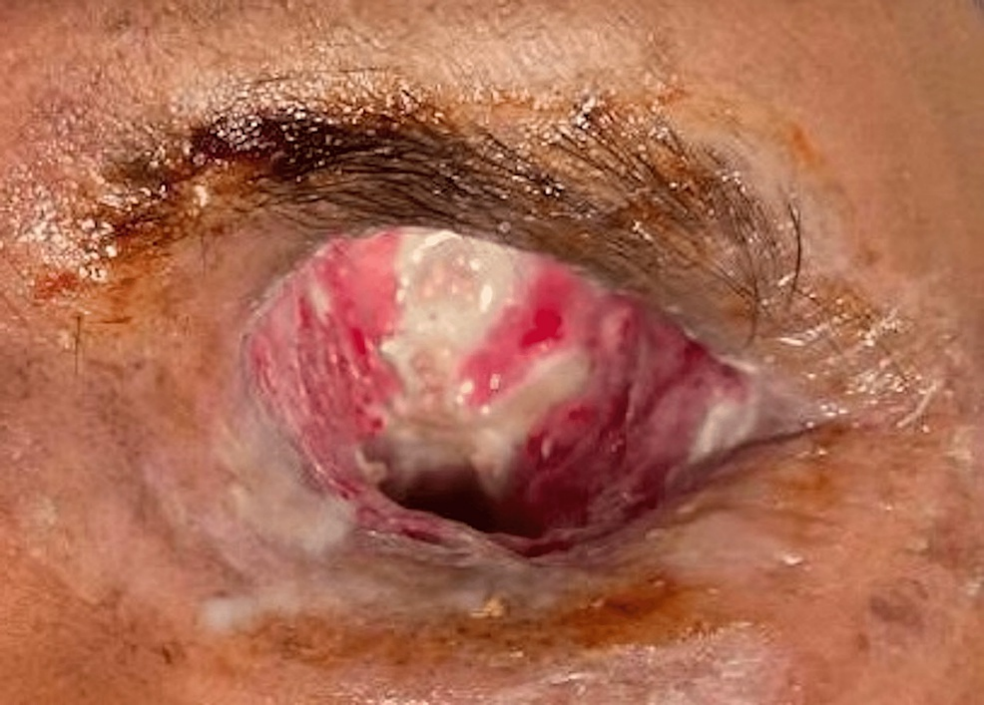
Exenterated left eye

The mortality rate was 20% (3/15) in patients treated with orbital exenteration. Out of these three patients, two were stage 4 with CNS involvement and one was stage 3d. Orbital exenteration is always done after histopathological confirmation of diagnosis (Figure 9).

The available options for cosmetic rehabilitation should be informed to the patients at this stage, which may help them accept this disfiguring but life-saving procedure. In our study, the survival rate was 100% if a patient presented with stage 1 ROCM and was minimum (45.45%) for patients with stage 4 ROCM. The stage at which the patient presents is critical in determining the outcome; hence, early diagnosis and management is the key to a favorable prognosis.

ROCM with cerebral involvement progresses rapidly with a reported death rate between 30% and 90% [8,13]. The overall mortality rate of 31% has been documented when associated with COVID-19 [24]. Another study from India shows that the overall mortality with COVID-19-associated ROCM is 14%, and disease progression is seen in 13% of cases. These results were stated to be likely to change over time as the patients were followed up. Our study stands out here with a follow-up of 12 months. We lost 16 patients to death in a follow-up of one year out of 41 deaths (39.02%). Based on our results, it is clear that the proposed staging corresponds to disease severity and survival outcome.

In the COSMIC study, in patients with a follow-up period of at least three weeks, 100% of the patients with stage 4 ROCM who had undergone orbital exenteration had stable residual or regressed lesions (p=0.03) [10]. Thus, according to them, surgery may not be a contraindication in patients with CNS involvement and may improve survival. However, since our study had a follow-up period of one year, we noticed that patients with stage 4 ROCM who were exenterated did not show favorable outcomes, while only one out of the remaining 13 exenterated patients with stage 3c and 3d ROCM died. These findings suggest that orbital exenteration may play a significant role in stage 3c and 3d disease. At the same time, a more conservative approach may be preferred in patients with disease stage 3b or better.

As far as visual outcome is concerned, 22 patients had vision between PL present and 6/60, and two had a visual outcome between 6/60 and 6/18. Out of 63 patients with ocular involvement, 15 patients died at the time of discharge. Therefore, we had 48 patients at one-month follow-up, out of which 14 were PL-negative (exenterated) and 10 had PR inaccurate. In the sixth month, 45 patients visited our center for follow-up. Thirteen were from the exenterated group and were PL-negative, and eight had PR inaccurate. At 12 months, again, we lost one patient from the exenterated group and one patient from the PR inaccurate group, making 12 PL-negative patients and seven with PR inaccurate visual outcomes. The rest of the final visual outcome was the same as that at one month.

Retrobulbar injection of amphotericin B at 3.5 mg/mL has effectively saved the eyes and lives of some patients with orbital mucormycosis [25-27]. However, more data is needed to assess its safety and potential for improving vision. Our study of 26 patients with minimal orbital disease who received retrobulbar amphotericin B found that 17 retained the same visual acuity, two showed improvement, five died, and two required exenteration but remained stable at one-year follow-up. Another study confirmed the efficacy of retrobulbar amphotericin B in patients with mild to moderate orbital mucormycosis [28]. This treatment may be beneficial in the early stages of the disease or in patients who cannot undergo surgery due to various factors.

## Conclusions

In conclusion, this research study represents a significant contribution to the understanding of post-COVID-19 rhino-orbital-cerebral mucormycosis (ROCM). With the largest number of patients from a single center and the longest follow-up duration, this study provides valuable insights into the management of this devastating fungal infection. The findings of this study emphasize the need for caution when using corticosteroids in patients with COVID-19, as they can increase the risk of mucormycosis in susceptible individuals. To prevent the occurrence of mucormycosis, it is crucial to identify and address risk factors, as well as maintain good glycemic and metabolic control in patients. The management of mucormycosis involves a combination of antifungal agents and surgical debridement. Additionally, the use of retrobulbar amphotericin B injections has shown promising results in salvaging the affected eye, which would otherwise be at risk of devastating damage caused by the fungus. Early diagnosis and aggressive treatment are paramount to achieving successful outcomes in patients with ROCM.

In advanced stages of the disease, orbital exenteration may be necessary to prevent further spread of the infection. However, in earlier stages, more conservative approaches can be effective. It is important to provide patient counseling for cosmetic rehabilitation, as ROCM can have significant aesthetic implications. Given the complex nature of ROCM, a multidisciplinary approach involving various specialists is crucial for optimal patient care. Collaboration between ophthalmologists, infectious disease specialists, endocrinologists, and other relevant healthcare professionals is necessary to ensure comprehensive and effective management of this condition. Overall, this study highlights the importance of early detection, appropriate treatment, and a multidisciplinary approach in combating post-COVID-19 ROCM. Further research and clinical studies are warranted to enhance our understanding and improve the outcomes for patients affected by this devastating fungal infection.

## Appendices

The proposed staging for rhino-orbital-cerebral mucormycosis (ROCM) is shown in Table 11. 

**Table 11:** Proposed staging for ROCM Reproduced with permission from Honavar SG. Code Mucor: Guidelines for the Diagnosis, Staging and Management of Rhino-Orbito-Cerebral Mucormycosis in the Setting of COVID-19. Indian Journal of Ophthalmology. 2021;69:1361-5 [29] ROCM: rhino-orbital-cerebral mucormycosis, MRI: magnetic resonance imaging, CT: computed tomography

Staging of ROCM	Symptoms	Signs	Primary assessment	Confirmation of diagnosis
Stage 1: Involvement of the nasal mucosa. 1a: Limited to the middle turbinate. 1b: Involvement of the inferior turbinate or ostium of the nasolacrimal duct. 1c: Involvement of the nasal septum. 1d: Bilateral nasal mucosal involvement.	Nasal stuffiness, nasal discharge, foul smell, epistaxis	Foul-smelling sticky mucoid or black-tinged, or granular or hemorrhagic nasal discharge, nasal mucosal inflammation, erythema, violaceous or blue discoloration, pale ulcer, anesthesia, ischemia, eschar	Diagnostic nasal endoscopy, contrast-enhanced MRI (preferred), or CT scan	Deep nasal swab or endoscopy-guided nasal swab or nasal mucosal biopsy for direct microscopy, culture, and molecular diagnostic, nasal mucosal biopsy for rapid histopathology with special stains
Stage 2: Involvement of paranasal sinuses. 2a: One sinus. 2b: Two ipsilateral sinuses. 2c: >Two ipsilateral sinuses and/or palate/oral cavity. 2d: Bilateral paranasal sinuses and involvement of the zygoma or mandible.	Symptoms in stage 1+ facial pain, facial edema, dental pain, systemic symptoms (malaise and fever)	Sign in stage 1+ unilateral or bilateral, localized or diffuse facial edema, edema localized over the sinuses, localized sinus tenderness	Diagnostic nasal endoscopy, contrast-enhanced MRI (preferred), or CT scan	Same as stage 1+ sinus biopsy for direct microscopy, culture, and molecular diagnostics and rapid histopathology
Stage 3: Involvement of the orbit. 3a: Nasolacrimal duct, medial orbit, vision unaffected. 3b: Diffuse orbital involvement (>1 quadrant or >2 structures), vision unaffected. 3c: Central retinal artery or ophthalmic artery occlusion or superior ophthalmic vein thrombosis; involvement of the superior orbital fissure, inferior orbital fissure, orbital apex, loss of vision. 3d: Bilateral orbital involvement.	Symptoms in stages 1 and 2 + pain in the eye, proptosis, ptosis, diplopia, loss of vision, infraorbital and facial V1/V2 nerve anesthesia	Sign in stage 1 and 2+ conjunctival chemoses, isolated ocular motility restriction, ptosis, proptosis, infraorbital nerve anesthesia, central retinal artery or ophthalmic artery occlusion, or superior ophthalmic vein thrombosis, V1/V2 nerve anesthesia, and feature of III, IV, and VI nerve palsy indicating orbital apex/superior orbital fissure involvement	Diagnostic nasal endoscopy, contrast-enhanced MRI (preferred), or CT scan	Same as stage 2+ orbital biopsy if indicated and if feasible (if the disease is predominantly orbital) for direct microscopy, culture, and molecular diagnostics and rapid histopathology
Stage 4: Involvement of the CNS. 4a: Focal or partial cavernous sinus involvement and/or involvement of the cribriform plate. 4b: Diffuse cavernous sinus involvement and/or cavernous sinus thrombosis. 4c: Involvement beyond the cavernous sinus, involvement of the skull base, internal carotid artery occlusion, brain infarction. 4d: Multifocal or diffuse CNS disease.	Symptoms in stages 1 and 3 + bilateral proptosis, paralysis, altered consciousness, focal seizures	Sign in stage 1-3 (some feature overlap with stage 3) + V1/V2 nerve anesthesia, ptosis, and feature of III, IV, and VI nerve palsy indicating cavernous sinus involvement; bilaterally of these sign with contralateral orbital edema with no clinicoradiological evidence of paranasal sinus or orbital involvement on the contralateral side indicating cavernous sinus thrombosis; hemiparesis, altered consciousness, and focal seizure indicating brain invasion and infarction	Diagnostic nasal endoscopy, contrast-enhanced MRI (preferred), or CT scan	Same as stage 3
